# Contribution of Social Isolation, Restraint, and Hindlimb Unloading to Changes in Hemodynamic Parameters and Motion Activity in Rats

**DOI:** 10.1371/journal.pone.0039923

**Published:** 2012-07-02

**Authors:** Darya Tsvirkun, Jennifer Bourreau, Aurélie Mieuset, Florian Garo, Olga Vinogradova, Irina Larina, Nastassia Navasiolava, Guillemette Gauquelin-Koch, Claude Gharib, Marc-Antoine Custaud

**Affiliations:** 1 UMR CNRS 6214 – INSERM 1083, Faculté de Médecine d’Angers, Université d’Angers, Angers, France; 2 Department of Human and Animal Physiology, M.V. Lomonosov Moscow State University, Moscow, Russia; 3 Centre National d’Etudes Spatiales, Paris, France; 4 ISOSTEO-LYON (Institut Supérieur d’Ostéopathie), Limonest, France; 5 Faculté de Médecine Lyon-Est, Physiologie, Lyon, France; 6 Associated French-Russia laboratory CaDyWEC (Cardiovascular Dysfunction induced by Weightlessness and Environmental Conditions), Angers, France; 7 Institute for Biomedical Problems Russian Academy of Sciences SSC, Moscow, Russia; 8 Explorations Fonctionnelles Vasculaires, CHU d’Angers, Angers, France; University of Otago, New Zealand

## Abstract

The most accepted animal model for simulation of the physiological and morphological consequences of microgravity on the cardiovascular system is one of head-down hindlimb unloading. Experimental conditions surrounding this model include not only head-down tilting of rats, but also social and restraint stresses that have their own influences on cardiovascular system function. Here, we studied levels of spontaneous locomotor activity, blood pressure, and heart rate during 14 days under the following experimental conditions: cage control, social isolation in standard rat housing, social isolation in special cages for hindlimb unloading, horizontal attachment (restraint), and head-down hindlimb unloading. General activity and hemodynamic parameters were continuously monitored in conscious rats by telemetry. Heart rate and blood pressure were both evaluated during treadmill running to reveal cardiovascular deconditioning development as a result of unloading. The main findings of our work are that: social isolation and restraint induced persistent physical inactivity, while unloading in rats resulted in initial inactivity followed by normalization and increased locomotion after one week. Moreover, 14 days of hindlimb unloading showed significant elevation of blood pressure and slight elevation of heart rate. Hemodynamic changes in isolated and restrained rats largely reproduced the trends observed during unloading. Finally, we detected no augmentation of tachycardia during moderate exercise in rats after 14 days of unloading. Thus, we concluded that both social isolation and restraint, as an integral part of the model conditions, contribute essentially to cardiovascular reactions during head-down hindlimb unloading, compared to the little changes in the hydrostatic gradient.

## Introduction

It is well established that exposure to microgravity results in dramatic reduction of general movement and loading in astronauts, which specifically leads to hypokinesia and hypodynamia. Together with fluid shift, hypovolemia and lack of hindlimb weight bearing significantly contribute to the development of cardiovascular deconditioning. Despite extensive cardiovascular research in the context of space flight, the mechanisms of postflight cardiovascular dysfunctions and complex countermeasures remain to be elucidated. Therefore there is a demand for animal ground models for the simulation of physiological and morphological consequences of space flight. Various modifications of hindlimb unloading and head-down tilt models in rats were described by Morey [1], which are predominantly used in the field. These models induce muscle atrophy and changes in bone structure as physiological consequences observed in humans after space flight or bed-rest [2]–[9]. Other physiological changes as synaptic plasticity changes have been described with this model [10]. In cardiovascular functions, rodent head-down tilt simulates cephalic fluid redistribution, hypovolemia, alters baroreflex function, and causes vessels structural as well as functional adaptations [11]–[13]. Data surrounding blood pressure (BP) and heart rate (HR) responses, however, remain controversial [14], [15]. Heterogeneity can be attributed to differences in animal tilting techniques, angle of tilt, suspension duration, blood pressure measurement protocols, recovery period duration after surgery, and control group choice [15]–[17].

In studies with hindlimb unloading, two experimental variables are particularly important – hindlimb unloading by itself and housing conditions. As a rule, all observed differences between the experimental groups are attributed to head-down tilting. However, restraint and social isolation are experimental parameters that have their own relative effects on the animals. To properly interpret cardiovascular changes induced by hindlimb unloading, a precise description of locomotor activity and documentation of heart rate and blood pressure changes in environmental conditions are imperative.

**Figure 1:**
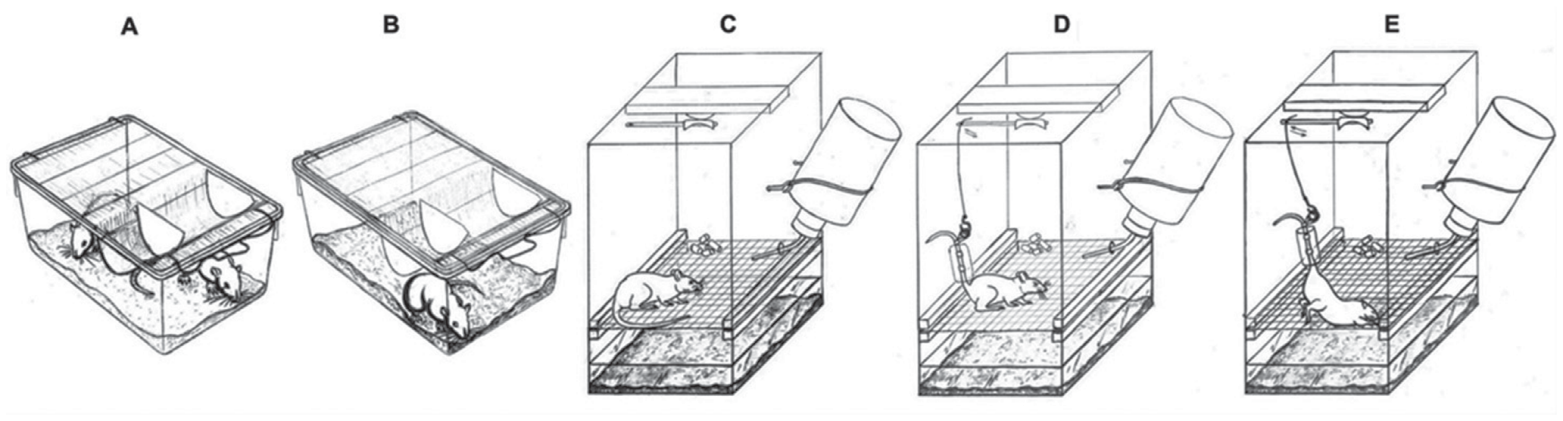
Design of the experimental cages. Control (**A**), isolated-control (**B**), isolated (**C**), attached (**D**), and unloaded (**E**) rats.

It is known that a sign of cardiovascular deconditioning in humans is post-flight orthostatic intolerance, which is accompanied by reduction of exercise capacity [18]–[20] and exaggerated heart rate responses. These findings have been well reproduced through ground-based human models of bed rest and dry immersion. Whether these responses occur in rats after hindlimb unloading is yet to be ascertained. Therefore, we sought to determine whether employing moderate exercise to characterize the extent of relative cardiovascular changes in rats is feasible.

In this study, we evaluated spontaneous locomotor activity (SLA), blood pressure, and heart rate changes that occurred as a result of 14 days of social isolation, restraint, and hindlimb unloading in rats. Our specific aims were to examine the influence of social isolation, restraint, and hindlimb unloading on spontaneous locomotor activity, to assess blood pressure and heart rate responses of conscious, free-moving rats during 14 days of social isolation, restraint, and hindlimb unloading. Finally, we evaluated the model for ground cardiovascular studies of weightlessness consequences.

## Materials and Methods

### Ethics Statement

All manipulations with animals were performed in accordance with US National Institutes of Health guidelines and European Community standards on the Care and Use of Laboratory Animals (Ministère de l’Agriculture, France, authorisation No 49072). This protocol has been approved by the regional ethic committee: Comité régional d’éthique pour l’expérimentation animale – Pays de la Loire, protocol No 2008.15.

### Animals

Male Wistar rats (5–6 weeks old) weighing 170–200 g were obtained from “Janvier,” Angers, France. Rats were housed under conditions of controlled temperature 24±2°C and 60% relative humidity. Animals were synchronised for a 12∶12 h light-dark cycle (light on at 0700 h, light off at 1900 h). Food and water were available *ad libitum*.

### Surgery

A telemetry system (Data Science International® - DSI, St Paul, MN, USA) was used to monitor blood pressure, heart rate, locomotor activity, and body temperature for conscious, freely moving rats. After 2–3 weeks of acclimatisation to laboratory conditions and daily handling, rats were surgically fitted with intraperitoneal radiotelemetry transmitters (TL11M2-C50-PXT, DSI) according to the recommendations of DSI and as previously described [21], [22]. Surgery was performed under isoflorane anaesthesia (2–4% in breathing air). Anesthetised rats received an intramuscular injection of Temgesic (buprenorphine, 0.1 mg/kg) to provide analgesia during surgeries. A blood pressure catheter was placed in the lower abdominal aorta and secured with surgical glue (3 M Vetbond™, USA). Its placement was verified with a radio receiver. ECG leads for lead II ECG recordings were placed and sutured subcutaneously as follows: the negative lead in the aria of the right shoulder, and the positive lead on the left side on the level of xiphoid space and caudal to the heart. A transmitter was secured in the abdomen by suturing the muscle wall using a non-absorbable suture. Rats recovered for 14 days and received 3 subcutaneous injections of antibiotic (Streptomycin, 40 mg/kg/d). Post-operative analgesia was provided by 40 mg/kg of Pediatric Ibuprofen (Advil®) in drinking water for 3 days post-surgery. Rats were housed in standard cages for laboratory animals (home cages) individually during the first 7 days of recovery. After this period, each rat was placed with a non-operated partner. Non-operated partners were chosen among the animals in the same cages in which operated rats were housed before the surgery (4 rats in a cage).

**Table 1:** Body weight and food consumption.

	Body weight (g)		Delta of body weight (g)	Food consumption (mg/day/100 g BW)
Groups	before experiment	end of experiment		
Control	398.9±12.8	445.9±13.5	47.0±6.0	5.2±0.2
Isolated-control	411.8±6.7	460.4±5.7	48.6±3.3	5.4±0.3
Isolated	419.1±11.4	463.3±9.9	44.2±4.0	5.8±0.5
Attached	383.3±10.5	412.4±11.0*	29.1±4.5*	5.4±0.1
Unloaded	402.7±8.2	398.1±8.7**	−6.1±5.1***	5.2±0.2

Values are group means for 7 days ± SEM, *P < 0.05, **P < 0.01 ***P < 0.001 *vs.* control group.

### Experimental Design and Experimental Groups

After the recovery period, rats remained in their home cages with non-operated partners. The cages were placed on receivers and baseline recordings of blood pressure waveform, ECG, and spontaneous locomotor activity in conscious rats were continuously performed using a telemetry data acquisition system (Data Science International® - DSI, St Paul, MN, USA) for 3 days.

Following 3 d of baseline recordings, all animals were assigned to 5 experimental groups (see Figure 1):

**Control** group rats (n = 7 – Control) were housed in pairs that were free to move in standard plastic cages with litter.
Socially-isolated rats comprised two groups. The **isolated-control** group (n = 6 – Isolated-control) rats were housed individually and allowed to freely move in standard plastic cages with litter. The **isolated** group (n = 7 – Isolated) rats were housed individually and allowed to freely move in the same cages as unloaded rats.
**Unloaded** rats (n = 6 – Unloaded) were housed individually in Plexiglass cages (30 cm×30 cm×45 cm, surface area 900 cm^2^) with grid floors. These animals were attached to suspension devices by tail harnesses, which enabled both hindlimb elevation to 40° above the cage floor and free movement of animal forelimbs. Attachment was performed under isoflorane anaesthesia. The tail was cleaned and degreased with medical ether. Half of the dorsal and ventral surfaces of the tail were covered by colophany solution and secured with an adhesive patch. The lateral sides of the tail remained uncovered for normal thermoregulation. The tail was then connected to a swivel placed on the top of the cage, which permitted free 360° rotation by the rat.
**Attached** rats (n = 7 – Attached) were housed individually in the same cages as the unloaded rats. The same tail harnesses were used as for the unloaded animals, but their hindlimbs were allowed to have full contact with the cage floor.

Rats were weighed twice a week during the periods of acclimatization for laboratory conditions, after transmitter implantation (from the third day after the surgery), and during the experimental period. Food was weighted and water volume was also marked to estimate food and water consumption during both baseline and experimental periods.

**Figure 2:**
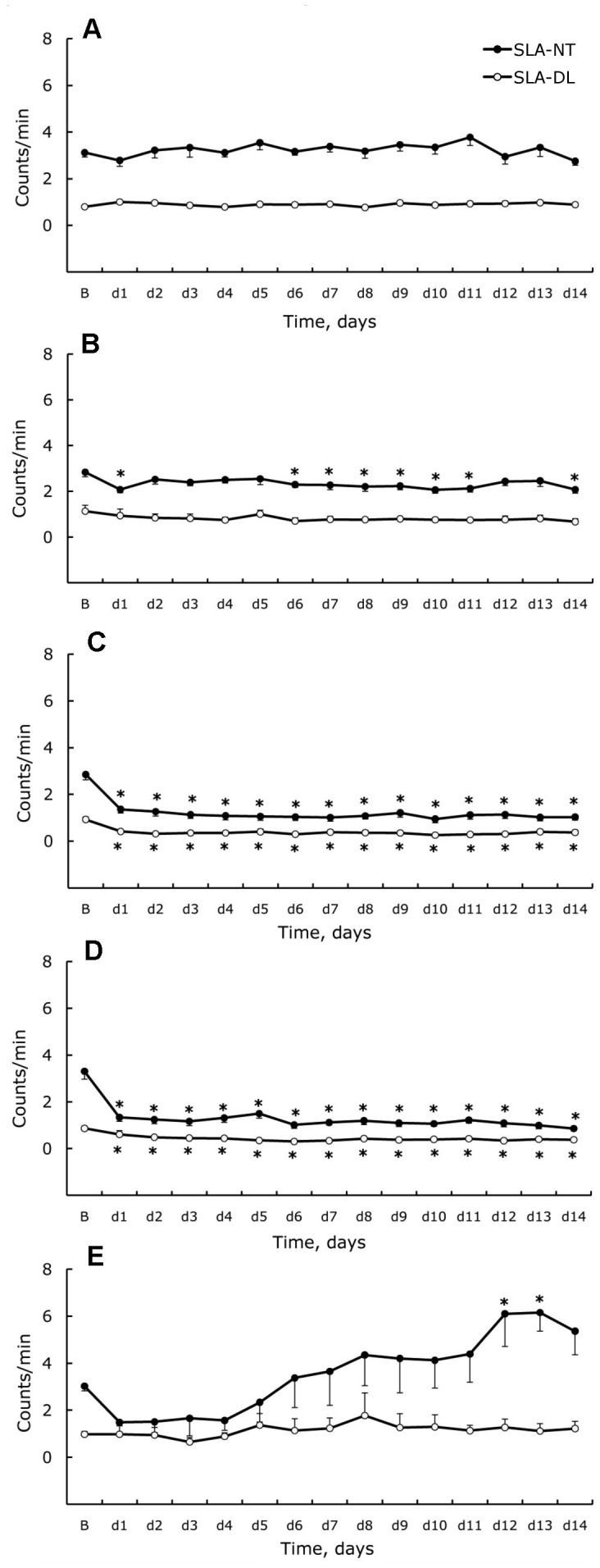
Nighttime (NT) and daylight (DL) spontaneous locomotor activity. Activity was measured in standard cages (BL) and during 14 days (d1–d14) for control (**A**), isolated-control (**B**), isolated (**C**), attached (**D**), and unloaded (**E**) rats. Data are given as a mean ± SEM. *- P < 0.05 *versus* basal level (BL).

### Data Acquisition and Analysis

The data were acquired and analysed using Dataquest A.R.T.™System (DSI, St Paul, MN, USA). BP and HR signals were recorded on eight animals simultaneously in continuous mode with a 2 s sampling period. The systolic (SBP) and diastolic (DBP) blood pressure, heart rate, and spontaneous locomotor activity were then assessed over an average of 30 min. For further statistical analysis, all measured variables were again averaged for 12 h and pooled for each day. Baseline recordings were the mean values for 3 d of recordings. Daytime (DT) values were acquired from 07∶00 h to 19∶00 h (light phase) and nighttime (NT) values were obtained from 19∶00 h to 07∶00 h (dark phase).

### Treadmill Running

After the postsurgical recovery period and before baseline recordings, all animals were habituated to treadmill running during 5 consecutive days on a motorised treadmill. Each rat began walking 8 m/min (0° incline), 5 min/day for 2 days, followed by 2 days at 10 m/min, and subsequently 1 day of 5 min running at 13 m/min. All trained animals then performed two tests of treadmill running with simultaneous mean arterial pressure (MAP) and heart rate monitoring. The first measurement of these variables took place after baseline recordings (PRE) and the second occurred after 14 d of respective treatment conditions (POST). Each of the two test exercises included recordings in the “home”-cage (Cage), 15 min of adaptation and equilibration on a stationary treadmill (TM 1), 5 min of running at 13 m/min, 0° incline (Ex), and 15 min of post-exercise recovery on a stationary treadmill (TM 2). Data are presented as an average over 5 min on each stage.

**Figure 3:**
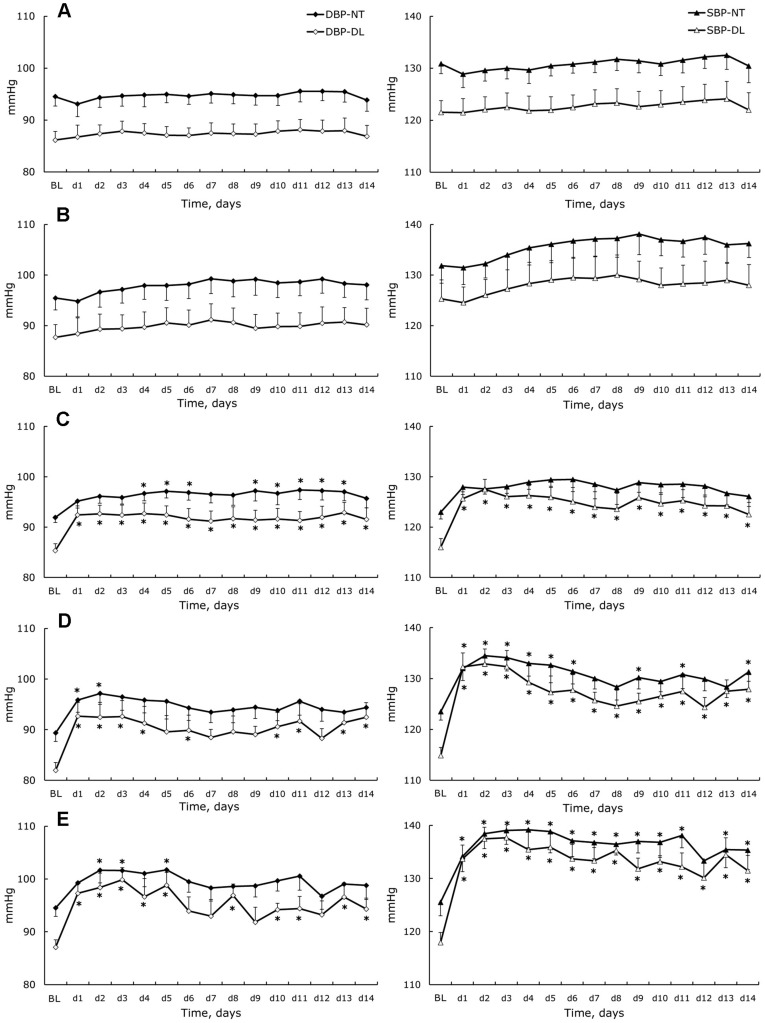
Diastolic and systolic blood pressure changes. Nighttime (NT) and daylight (DL) diastolic (left) and systolic (right) blood pressure measured in standard cages (BL) and during 14 days (d1–d14) for control (**A**), isolated-control (**B**), isolated (**C**), attached (**D**), and unloaded (**E**) rats. Data are given as a mean ± SEM. *- P < 0.05 *versus* basal level (BL).

**Figure 4:**
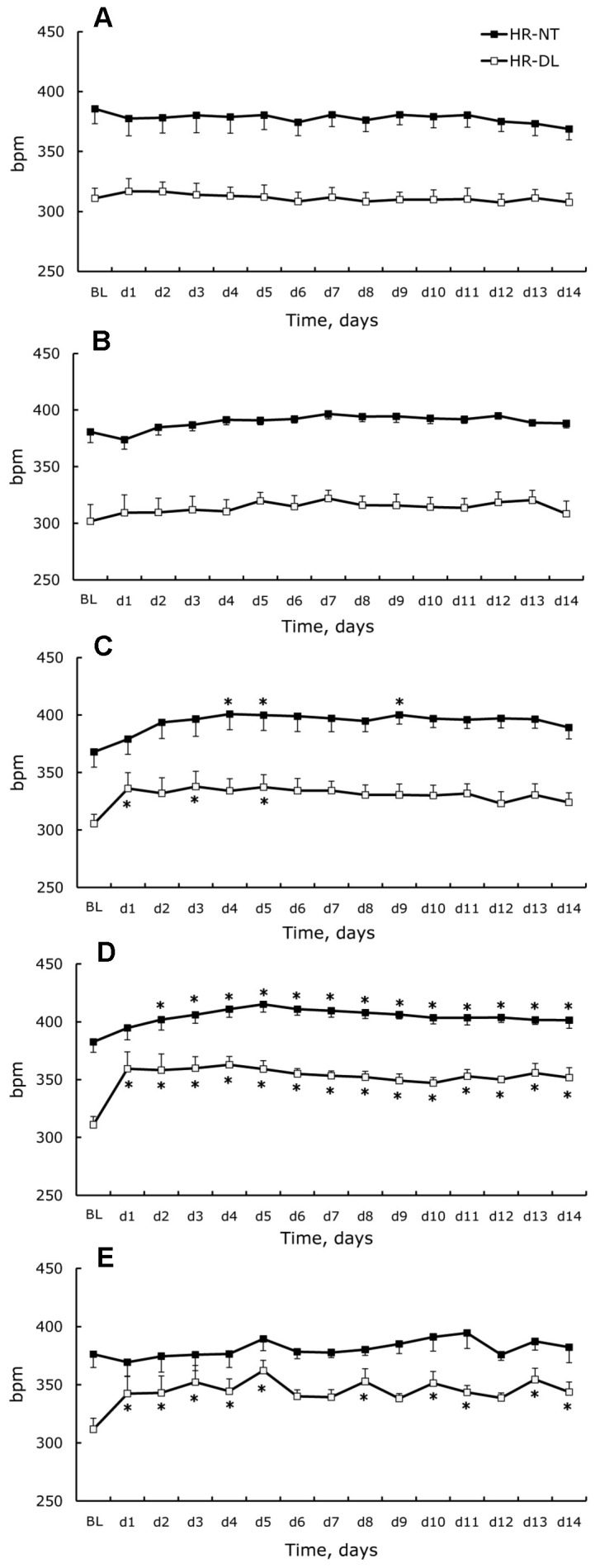
Heart rate changes. Nighttime (NT) and daylight (DL) heart rate measured in standard cages (BL) and during 14 days (d1–d14) for control (**A**), isolated-control (**B**), isolated (**C**), attached (**D**), and unloaded (**E**) rats. Data are given as a mean ± SEM. *- P < 0.05 *versus* basal level (BL).

### Data Evaluation and Statistical Analysis

All values are presented as a mean ± SEM and *P* < 0.05 was considered statistically significant. Data were analyzed by one-way repeated measures of analysis of variance (ANOVA) to compare blood pressure, heart rate, and locomotor activity values between baseline and the 14 days experimental period. ANOVA was followed by a Fisher LSD post-hoc test where appropriate for further comparison of all pairs. For the treadmill running tests statistical analysis was performed in a parallel manner. The values between both the daylight and nighttime periods for each of five experimental groups were also compared, but to improve readability, they are not discussed in the text.

**Figure 5:**
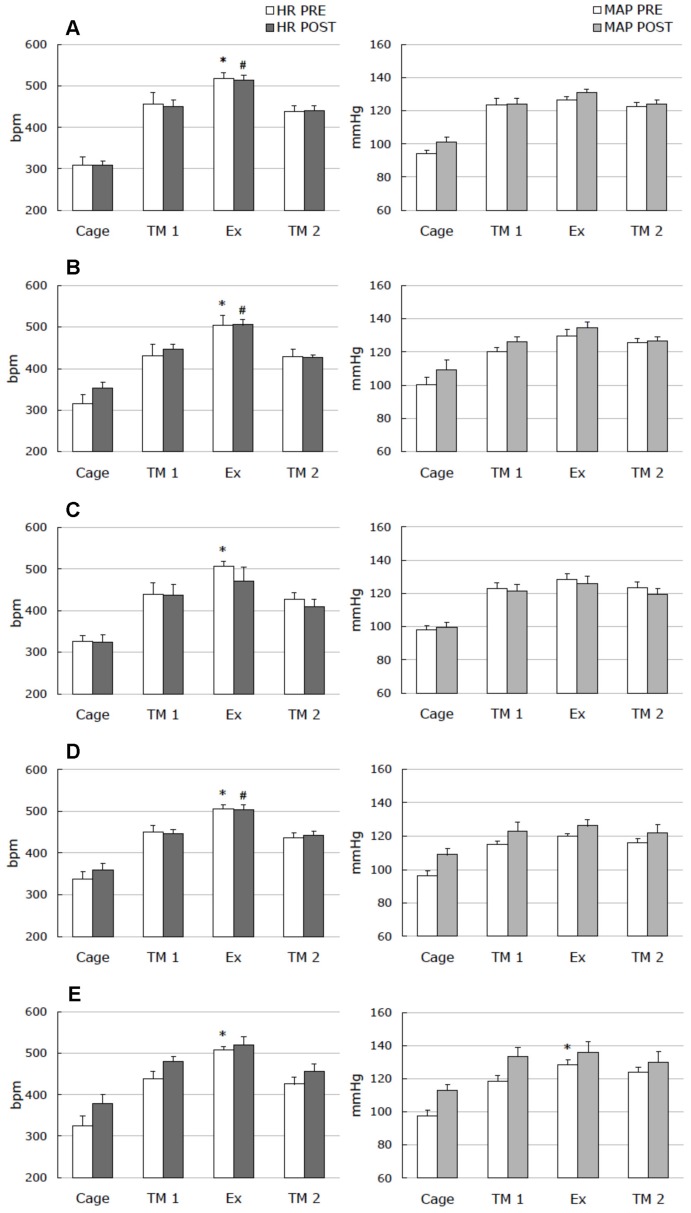
Heart rate and blood pressure during treadmill running. Heart rate (left) and mean arterial pressure (right) during a treadmill running test before (**PRE**) and after (**POST**) 14-days of control (**A**), isolation-control (**B**), isolation (**C**), attachment (**D**), and unloading (**E**). Cage: in experimental cage. TM1: in the treadmill cage, at rest, before exercise. EX: during treadmill running. TM2: in the treadmill cage, at rest, after exercise. Data are given as a mean ± SEM. *and # - P < 0.05 *versus* respective TM1 level.

## Results

### Body Weight, Temperature and Food Consumption

The body weight was similar in all groups at the beginning of the experiment (Table 1). During the subsequent 14 d of experimentation, all of the animals gained weight, except the unloaded rats. Unloaded and attached animals weighted less than rats in the control group by the end of the 14 d period. Average daily food consumption was similar throughout the experiment in all groups (Table 1).

Night temperature was significantly higher than respective daylight values in traditional cages (baseline) for animals of all the experimental groups. This difference reflects normal diurnal fluctuations and remained unchanged throughout the experiment. We only observed a short-term increase of daytime temperature for unloaded (day 1 and day 2 of the experiment) and attached (day 1) rats.

### Changes of Spontaneous Locomotor Activity

Daylight and nighttime values of SLA during the experiment are shown in Figure 2 for the 5 experimental groups. Basal levels of SLA measured in standard cages showed normal diurnal fluctuations with the level higher in nighttime. In control rats, 24 h patterns of SLA changes during 12∶12 h light-dark cycle remained undamaged for 14 d. Social isolation itself (isolated control) did not cause any substantial changes of activity or rhythm during the light-dark cycle. Statistically significant differences were observed only in NT-SLA on day 1, days 6–11, and day 14. Attachment and social isolation in the same cages than hindlimb unloading resulted in a very similar and dramatic decrease of SLA that began on day 1 and continued throughout the experiment. In both experimental groups, nighttime activity was similar to the basal daytime value. Diurnal fluctuations of SLA were unchanged and activity was higher at night. Hindlimb unloading caused a significant perturbation of SLA during the NT period, while DT activity was constantly low and similar to control. The changes of NT-SLA were biphasic, whereby decreased activity was observed in the rats during the first 4 days of unloading followed by a significant increase of motor activity. Differences from basal level by day 12 and 13 were statistically significant.

### Changes of Blood Pressure and Heart Rate

DT and NT values of blood pressure (SBP, DBP) and heart rate are shown in Figure 3 and 4. In full accordance with locomotor activity basal levels, the SBP, DBP, and HR measured in standard cages were higher at night for all experimental groups. In control rats, neither changes in the cardiovascular parameters nor alterations of circadian variations were observed during the 14 d experiment. Social isolation itself did not lead to significant perturbations of measured cardiovascular parameters or changes in rhythmicities. However, two weeks of social isolation under conditions of unloading (isolated group) caused a significant increase of SBP and DBP during the light phase starting from day 1. Slight tachycardia was observed a few days after isolation. In the attached group, despite a dramatic decrease of SLA, we observed a clear rise of SBP, HR, and DT-DBP. Hindlimb unloading also resulted in substantially increased SBP over 24 h and increased daylight DBP. For nighttime DBP, there was a tendency to increase, but significant differences from basal level were only documented on days 2, 3, and 5. HR also increased during daylight only.

### Treadmill Running

Heart rate and mean arterial pressure during running tests are shown in Figure 5. In all experimental groups, HR and MAP significantly rose when animals were placed on the treadmill, and no reaction differences were observed between PRE and POST tests. Five minutes of running caused a significant supplementary increase of HR compared to the level achieved on the stationary treadmill in all groups during the PRE test. None of the experimental conditions used in the study (control, isolation, isolation control, attachment, and unloading) induced significant changes in this parameter (P>0.05).

## Discussion

In this study, we report the following observations:

1. Social isolation and restraint induced persistent physical inactivity, while unloading in rats resulted in initial inactivity followed by normalization and increased locomotion after one week.
2. Hindlimb unloading, restraint stress, and social isolation caused significant perturbations in blood pressure and heart rate levels in rats.
3. We detected no augmentation of tachycardia during moderate exercise in rats after 14 d of unloading.

### Spontaneous Locomotor Activity

We observed that hindlimb unloading, restraint stress, and social isolation in identical housing conditions have a significant effect on spontaneous locomotor activity in rats. Rats are nocturnal animals, which is a trait that was observed in the control group, as motility was four times higher during the night than during the day throughout the experimental period. Isolation is abnormal for rodents, since they are naturally social. Various behavioral tests have shown that prolonged individual housing of adult rats produces depression and increases anxiety-like behavior, reluctance of locomotor and explorative activity, delays habituation to novel conditions, and increases the response to stress [23]–[27]. We observed a mild decrease of general locomotor activity during the nighttime with no changes during the light phase when animals were rested and activity was low in the isolated-control group. We also considered that a depressive-like behavior stemmed from social stress influences through cohabitant loss and absence of normal social relationships. Chronic stress was shown to be associated with reduced locomotor activity and spontaneous wheel running [28], [29], which supports our theory. Comparing the two groups of isolated animals, we ultimately deduced a cage effect. For unloading, we used transparent grid floor cages [30]. Barriers between the cages prevented animals from seeing each other while sparing olfactory and auditory contact. Social isolation in the cages for hindlimb unloading had appreciable influences, causing dramatic decreases in activity during both day and night. Such hypokinesia was present throughout the 14 d experiment. This observation related conditions of unloading to perturbations in a rat’s normal daily life, which includes social relation and nest building. Changes in these variables are stressful for animals despite the generally accepted view that rats quickly grow accustomed to experimental conditions and the fact that neither loss in body weight nor hyperthermia was documented in our isolated group.

Rats from the attached group were exposed to social stress and restraint. Restraint did not change the profile of spontaneous locomotor activity compared to the isolated group, and attached animals showed a very low level of activity throughout a 24 h period, which we also considered as a depressive-like period. We propose that in attachment conditions, contributions of social stress and cage type are most influential in animal behavioral changes and that mild supplementary restriction was too trivial to further aggravate the situation.

Surprisingly, hindlimb unloading produced biphasic changes of motor activity. We observed clear signs of hypokinesia for only the first few days during the nighttime when rats are normally active. Daytime activity remained low and unchanged, similar to the control or isolated control groups. A tendency for decreased nighttime motor activity during the first week was replaced by stabilization and a further increase in the number of movements during the second week of unloading. When compared to isolated and attached groups, the hypokinesia observed in the beginning of the experiment was related to social stress and restraint. Further activation can be attributed to the action of unloading itself, since neither social isolation nor restraint caused an increase in spontaneous locomotor activity. This observation is important, since the model of hindlimb unloading is traditionally associated with hypokinesia, which affects humans during spaceflight. As shown by our experiments, this model is applicable only in reference to individual muscle groups in the context of muscle disuse, and remains unsuitable to explain cardiovascular changes. The decrease in body weight is usually explained as a result of muscle atrophy during hindlimb unloading [31]–[33]. However, the increase in locomotor activity during the second phase of unloading might also participate in the body mass reduction observed in this group. This result is in accordance with the increase in total energy expenditure reported in 14 day unloaded rats [34]. To our knowledge, no study has assessed general motor activity during hindlimb unloading, however we found one reference describing continuous activity measurements during 7 d of head-down tilt in tubular cages [15]. The authors also reported a reduction of nighttime somatomotor activity by 60% from the value obtained in traditional cages without significant perturbations during daylight. Considering the difference in the head-down tilting methods (hindlimb unloading and tilt in tubular cages), these data are consistent with the data in our study, specifically nighttime hypokinesia during the first week of unloading. Raffai and coauthors hypothesized that such an effect is a result of head-down body position, in which non-specific, non-gravitational stress was absent [15].

### Blood Pressure and Heart Rate Monitoring

In the present study, we observed essential changes of cardiovascular parameters (blood pressure and heart rate) in different experimental conditions associated with hindlimb unloading. In the control group, rats housed in pairs showed no changes of BP and HR throughout the 14 d experiment. Moreover, the rhythm of BP and HR over 24 h remained typical for nocturnal animals. This confirms that the general housing conditions were unchanged throughout the experiment and did not cause perturbation of everyday life or specific stress. Social isolation in the standard cages resulted in no changes in HR. No significant changes were observed in blood pressure when compared to base levels. However, the ANOVA analysis revealed a significant effect of the time on nighttime systolic and diastolic blood pressure, and we saw a tendency for both parameters to increase throughout the experiment. Similar to spontaneous locomotor activity, social isolation in the cages during hindlimb unloading caused notable changes in cardiovascular parameters, with the most significant increase occurring in blood pressure during daylight. Social isolation in individual cages causes behavioral changes in rats and can be used as a model for psychosocial-stress, which induces reversible hypertension accompanied by cardiovascular structural changes, such as left ventricular and aorta hypertrophy [35]–[39]. An increase in blood pressure can be reversed by treatment with β-adrenoreceptor antagonists, and is preventable by pretreatment [35]. We hypothesize that housing in conditions of social isolation supplemented with disturbances in the usual behavior of rats results in continuous mild stress and sympathetic system activation. In support of this hypothesis, we observed significant perturbations in 24 h blood pressure variability with marginal pressure fall during daylight in the isolated group – a non-dipper pattern that is partly due to vascular sympathetic activation that is also maintained at rest [40], [41]. It should be noted that in all studies listed, either small individual cages or individual glass metabolic cages with a grid floor and 380 cm^2^ surface area were used for isolation. Therefore, it is possible that such conditions simultaneously promote social stress and restraint compared to standard cages. Our observations in the group of attached rats are in accordance with established data, even when using cages with a greater surface (900 cm^2^). Attached rats exposed to conditions of social isolation in special cages with supplementary restraint stress showed increased blood pressure during the daylight phase with a non-dipper pattern, accompanied by a diurnal heart rate increase. The most significant changes were observed in SBP. A rise in SBP was observed immediately after restraint, which gradually decreased during the first 7 d of the protocol and reflected some type of adaptation to restraint. If we consider changes in the isolated group, we can also postulate sympathetic activation. In support of this hypothesis, we observed that isolation and restraint (attachment) was accompanied by a dramatic decrease of daily activity. It has been reported that sedentary animals show enhanced sympathoexcitation and altered sympathetic control of blood pressure [42], [43]. Our unloaded rat data are in accordance with what is cited in the literature, even considering differences in the methods of cardiovascular parameter measurements [17], [44], [45]. Unloading caused very similar changes in blood pressure when compared to the group of attached animals, where an increase of diastolic pressure and dramatic rise of SBP was accompanied by a significant perturbation of the day-night cycle. Surprisingly, unloaded animals’ heart rate did not increase at night. We are unable to fully explain such a difference in heart rate changes between day and night in these rats, but we hypothesize that the fluid shift induced by the head-down tilt position in unloaded rats may blunt the increase of heart rate during the nighttime when the rats are active. It should be highlighted that signs of fluid shift are well documented only in the “acute” phase of head down tilt induced by hindlimb unloading – an increase of subcutaneous tissue fluid pressure in the neck after 48 h of head-down tilt in rats [46], increase in the central venous pressure with duration dependent on the unloading angle [47], increase in the atrial natriuretic factor concentration after 2 h of unloading [48], and large but short-term post-antiorthostatic tilt increase in intra-cerebroventricular pressure followed by a moderate decrease [49], [50]. Simultaneous and continuous increases of blood pressure and heart rate in spite of important physical inactivity can be attributed to loss of hindlimb weight bearing and to the perturbation in the adrenocorticotropic axis as a result of sustained psychomotor strain during hindlimb unloading [49]. The same phenomenon may be responsible for the non-dipper pattern of blood pressure that was also observed in the unloaded group.

### Blood Pressure and Heart Rate Changes During the Treadmill Running

We failed to observe augmented tachycardia during exercise after two weeks in the unloaded group as well as in the other experimental groups. Although we used relatively moderate exercise methods, our results agree with Overton et al. [33], who applied more intense treadmill running after hindlimb unloading. They also observed no significant heart rate or arterial pressure changes and showed that an increase in heart rate during the exercise was not exaggerated by a head-down position at day 9. Similar results were also observed in head-down tilted and horizontally suspended rats. In our work, animal placement on a stationary treadmill initially caused a rise of pressure and heart rate. We therefore hypothesize that the “emotional” component may have a more important contribution to the cardiovascular response than exercise itself. By incorporating this finding into our result for continuous monitoring of heart rate and blood pressure in different experimental conditions, we hypothesize that the contributions of social stress, restraint, and lack of weight bearing to cardiovascular changes in the model are more essential than moderate hydrostatic pressure differences.

### Study Limitations

Considering that the DSI system only provides relative measurements of locomotor activity, we realized supplementary methodical experiment. This additional measurement aimed to avoid an error due to elevation of the hindlimbs and transmitter movement away from the cage floor. Four female rats weighing 230–250 g were surgically fitted with DSI transmitters as described in the Materials and Methods”. Rats remained in standard cages for the duration of the experiment. After the postsurgical recovery period, the cages were placed on receivers. Continuous recordings of SLA and signal strength were implemented for 7 d. The cages were then raised up to 19 cm over the receivers and recordings were continued for an additional 7 d. The increase in cage height resulted in an expected decrease of signal strength (28.1±0.1 vs. 24.5±0.1 MKUs), but SLA remained unchanged (daylight: 1.5±0.3 vs. 1.2±0.1 counts/min, respectively; nighttime: 3.2±0.1 vs. 3.4±0.1 counts/min, respectively). Therefore, measurement of SLA using DSI telemetry system can be implemented in our model of hindlimb unloading.

### Conclusion

In conclusion, both social isolation and restraint stress, as part of unloading model conditions, induce persistent physical inactivity. An increase in motor activity during the second week of hindlimb unloading, however, is a limit of the model, as physical inactivity is an important factor of cardiovascular deconditioning development. Social isolation and restraint stress causes complex changes in heart rate and blood pressure, which mimics unloading. After 14 d of hindlimb unloading, rats do not demonstrate augmented tachycardia during treadmill running, which is a key feature of cardiovascular deconditioning in humans. Cardiovascular changes observed in the hindlimb unloading model are commonly interpreted as a fluid shift consequence, especially for vascular changes. We must note that the modification of hydrostatic pressure in the thoraco-cephalic portion of the rat body in response to the head-down position is very low (few mmHg) and temporary. These local blood pressure changes induced by hindlimb unloading appear to be negligible compared to changes in systemic blood pressure, which is an important variable when analyzing vascular remodeling as a result of unloading.
